# Voluntary activation of the diaphragm after inspiratory pressure threshold loading

**DOI:** 10.14814/phy2.15575

**Published:** 2023-01-25

**Authors:** Andrew H. Ramsook, Michele R. Schaeffer, Reid A. Mitchell, Satvir S. Dhillon, Kathryn M. Milne, Olivia N. Ferguson, Joseph H. Puyat, Michael S. Koehle, A. William Sheel, Jordan A. Guenette

**Affiliations:** ^1^ Department of Physical Therapy, Faculty of Medicine The University of British Columbia Vancouver British Columbia Canada; ^2^ Centre for Heart Lung Innovation, Providence Research The University of British Columbia and St. Paul's Hospital Vancouver British Columbia Canada; ^3^ Division of Respiratory Medicine, Faculty of Medicine The University of British Columbia Vancouver British Columbia Canada; ^4^ Centre for Health Evaluation and Outcome Services, Providence Research The University of British Columbia and St. Paul's Hospital Vancouver British Columbia Canada; ^5^ School of Kinesiology, Faculty of Education The University of British Columbia Vancouver British Columbia Canada; ^6^ Department of Family Practice, Faculty of Medicine The University of British Columbia Vancouver British Columbia Canada

## Abstract

After a bout of isolated inspiratory work, such as inspiratory pressure threshold loading (IPTL), the human diaphragm can exhibit a reversible loss in contractile function, as evidenced by a decrease in transdiaphragmatic twitch pressure (P_DI,TW_). Whether or not diaphragm fatigability after IPTL is affected by neural mechanisms, measured through voluntary activation of the diaphragm (D‐VA) in addition to contractile mechanisms, is unknown. It is also unknown if changes in D‐VA are similar between sexes given observed differences in diaphragm fatigability between males and females. We sought to determine whether D‐VA decreases after IPTL and whether this was different between sexes. Healthy females (*n* = 11) and males (*n* = 10) completed an IPTL task with an inspired duty cycle of 0.7 and targeting an intensity of 60% maximal transdiaphragmatic pressure until task failure. P_DI,TW_ and D‐VA were measured using cervical magnetic stimulation of the phrenic nerves in combination with maximal inspiratory pressure maneuvers. At task failure, P_DI,TW_ decreased to a lesser degree in females vs. males (87 ± 15 vs. 73 ± 12% baseline, respectively, *p* = 0.016). D‐VA decreased after IPTL but was not different between females and males (91 ± 8 vs. 88 ± 10% baseline, respectively, *p* = 0.432). When all participants were pooled together, the decrease in P_DI,TW_ correlated with both the total cumulative diaphragm pressure generation (*R* ^2^ = 0.43; *p* = 0.021) and the time to task failure (TTF, *R* ^2^ = 0.40; *p* = 0.30) whereas the decrease in D‐VA correlated only with TTF (*R* ^2^ = 0.24; *p* = 0.041). Our results suggest that neural mechanisms can contribute to diaphragm fatigability, and this contribution is similar between females and males following IPTL.

## Abstract 2

A loss in voluntary activation after a task can be a marker of skeletal muscle fatigue. The diaphragm, the primary muscle of inspiration, exhibits a loss in voluntary activation following isolated inspiratory muscle work. When matched for total cumulative respiratory pressure generated throughout a task, males and females exhibit a similar decline in diaphragm voluntary activation.

## 1 INTRODUCTION

During dynamic fatiguing tasks, such as whole‐body exercise (Johnson et al., 1993), resistive breathing (McKenzie et al., 1997), or inspiratory pressure threshold loading (IPTL) (Geary et al., 2019; Welch et al., 2018a), the human diaphragm can experience a loss in contractile function, resulting in a temporary loss in the diaphragm's ability to generate maximal pressure. A temporary loss in a muscle's ability to generate maximal force or pressure after a task can be described as performance fatigability. In addition to a decline in pressure generation, voluntary activation can also be used to measure performance fatigability (Enoka & Duchateau, 2016). Previous research examining diaphragm fatigability has focused on the decrease in contractile function, while changes in diaphragm voluntary activation (D‐VA) have received less attention. The addition of D‐VA measurements would provide a more comprehensive examination of how neural processes contribute to fatigability and possibly, exercise performance. Recently, it has been shown that after sustained inspiratory efforts, male participants show a decrease in overall inspiratory muscle voluntary activation (Luu et al., 2020). However, the study by Luu et al. (2020) only included male participants, and therefore, it is unknown whether a decrease in voluntary activation can contribute to performance fatigability in female participants. Given the known sex differences in skeletal muscle fatigue (Hunter, 2014) and, specifically respiratory physiology (Dominelli & Molgat‐Seon, 2022), it stands to reason there may be an effect of sex on D‐VA after periods of high respiratory work.

There is a noted sex difference in diaphragm fatigability in humans. In general, females are less fatigable than their male counterparts; however, this varies based on the type of task performed (Hunter, 2016). In the diaphragm, after high‐intensity cycling (Guenette et al., 2010; Welch et al., 2018b) and isolated inspiratory work (Welch et al., 2018a), females develop less fatigue than males. However, these studies are limited to investigations of contractile function, measured through changes in transdiaphragmatic twitch pressure (P_DI,TW_) and did not measure the D‐VA. Furthermore, the inspiratory muscle metaboreflex, a reflex thought to be governed by mechano‐ and metabo‐sensitive group III/IV afferent feedback (St Croix et al., 2000) appear to be blunted in females performing inspiratory work (Geary et al., 2019; Smith et al., 2016; Welch et al., 2018a). Feedback from group III/IV afferents has been shown to promote the loss of voluntary activation in locomotor muscles after cycle exercise (Sidhu et al., 2017). Given this blunted inspiratory muscle metaboreflex response in females compared to males, it is possible that the loss of D‐VA may be less in females as well. Recently, we have shown that healthy, young males exhibit a greater loss in D‐VA compared to females after high‐intensity cycle exercise (Ramsook et al., 2022). However, this study focused on whole‐body exercise, a task where the active muscles must compete for a finite cardiac output (Calbet et al., 2007). It remains unknown whether sex differences in D‐VA would be present during isolated inspiratory muscle work designed to induce diaphragm fatigue, when cardiac output can be dedicated to serving the needs of the respiratory muscles to meet the oxygen cost of breathing. Accordingly, the primary purpose of this study was to investigate whether D‐VA changes after a single bout of IPTL in healthy adults. The secondary purpose of this study was to determine whether changes in D‐VA differ between males and females. We hypothesized that IPTL would result in a decrease in D‐VA from baseline in males and females, and that the decrease in D‐VA would be greater in males compared to females.

## 2 METHODS

### 2.1 Participants

Young (age 19–31 years), healthy never smokers with no symptoms or history of cardiovascular, respiratory, or musculoskeletal disease, and normal pulmonary function (*i.e.,* forced expiratory volume in 1 s (FEV_1_) to forced vital capacity ratio >0.70 and FEV_1_ ≥ 80% predicted) were included in this study. Females were tested randomly throughout their menstrual cycle and were not excluded if they were using oral contraceptives or an intrauterine device. Many participants (M:9, F:10) also participated in a previous study in our laboratory, which examined changes in D‐VA after whole‐body exercise (Ramsook et al., 2022). The current study represents a *de novo* research question, and no values have been reported in the aforementioned study.

### 2.2 Experimental overview

Participants reported to the laboratory on two separate occasions separated by at least 48 h. Participants were instructed to avoid caffeine for 8 h and exercise for 24 h prior to each testing session. Session 1 involved anthropometric measurements and pulmonary function tests (Vmax Encore 229 with V62J Autobox; CareFusion, CA, USA), which were performed in accordance with standard recommendations (Miller et al., 2005; Wanger et al., 2005). Inspiratory muscle strength was measured using a semi‐occluded mouthpiece connected to a calibrated differential pressure transducer (DP15‐34; Validyne Engineering, CA, USA) as recommended (Laveneziana et al., 2019). Pulmonary function and inspiratory muscle strength results were presented in absolute units and as a percentage of predicted values (Black & Hyatt, 1969; Gutierrez et al., 2004; Tan et al., 2011). Participants were then familiarized with the scales used to record dyspnea during IPTL as well as the IPTL protocol itself. Session 2 was the primary testing session where participants engaged in IPTL until task failure. Diaphragm contractile function and voluntary activation were measured during session 2 only.

### 2.3 Inspiratory muscle electromyography (EMG)

Crural diaphragm EMG (EMG_DI_) was measured using a multipair esophageal electrode catheter (Guangzhou Yinghui Medical Equipment Co. Ltd, Guangzhou, China), which was positioned based on the strength of the EMG_DI_ signal as previously described (Luo et al., 2008). EMG of the scalenes (EMG_SCA_) and sternocleidomastoid (EMG_SCM_) muscles were collected by placing bipolar surface electrodes (Dual EMG Wet Gel Ag/AgCl electrodes; Noraxon, AZ, USA) at the midpoint between the mastoid process and medial clavicle along the long axis of the sternocleidomastoid for EMG_SCM_, and at the level of the cricoid process within the posterior triangle of the neck for EMG_SCA_ with a wireless surface electromyography system (MyoSystem 1400A, Noraxon, AZ, USA). Raw EMG_DI_ signals were collected using an amplifier (bio‐amplifier model RA‐8, Guangzhou Yinghui Medical Equipment Co. Ltd.), converted from analog to digital (Power Lab 16/35, ADInstruments, CO, USA) and collected using LabChart Pro software (v8.1.19, ADInstruments). All EMG signals were sampled at 10 kHz. EMG_DI_ was also used to assess the parameters of the compound muscle action potential (CMAP). A custom MATLAB (R2021a; MathWorks, MA, USA) script was used to process raw EMG_DI_, filtered through a fourth order Butterworth filter (cut‐off frequencies: 20–500 Hz) and rectified to calculate CMAP peak‐to‐peak amplitude. Peak‐to‐peak amplitude was calculated as the sum of the two rectified peaks. CMAP onset was defined as the first point before the first peak that exceeded a threshold, calculated as the mean of the rectified EMG signal 40 milliseconds before the stimulation +2 standard deviations. CMAP offset was defined as the first point after the second rectified peak that fell below the same threshold, and CMAP duration was the difference between onset and offset. All EMG data were visually inspected and verified. Twitches were excluded if the CMAP signal was contaminated by cardiac artifact. EMG was also used to measure inspiratory muscle activation during IPTL. These EMG signals were filtered using a band‐pass filter (20–500 Hz) and converted to root‐mean square calculated using a 100‐millisecond moving average window. EMG_DI_ selections were performed manually, breath‐by‐breath, to avoid sections with cardiac artifact. EMG data during IPTL are presented relative to the maximal EMG of each inspiratory muscle achieved during a maximal inspiratory maneuver (*e.g.,* inspiratory capacity, maximal inspiratory pressure, or maximal voluntary ventilation maneuver, whichever was greatest).

### 2.4 Respiratory pressures

The catheter used to record EMG_DI_ was also used to measure gastric pressure (P_GA_). Once the P_GA_ balloon was placed, based on the strength of the EMG_DI_ signal (Luo et al., 2008), the balloon position within the stomach was confirmed via cough and sniff maneuvers. In addition, a second balloon catheter was used to measure esophageal pressure (P_ES_) (Adult esophageal balloon catheter, no. 47–9005; Cooper Surgical, CT, USA). The position of the esophageal catheter was verified via the occlusion test (Baydur et al., 1982) and monitored throughout the procedures to ensure the catheter did not move. Mouth pressure (P_MO_) was measured through a port in the mouthpiece. Catheter derived pressures and P_MO_ measurements were connected to calibrated differential pressure transducers (DP15‐34; Validyne Engineering, CA, USA). Transdiaphragmatic pressure (P_DI_) was calculated as the difference between P_GA_ and P_ES_. Pressure–time product of the diaphragm (PTP_DI_) was calculated by the numerical integration of an ensemble‐averaged P_DI_ trace during inspiration over a 30 s period during each minute of IPTL multiplied by the breathing frequency during that minute.

### 2.5 Inspiratory pressure threshold loading

Participants breathed on a custom‐built IPTL device described previously (Boyle et al., 2020). Participants inspired with sufficient negative pressure to lift a weighted plunger to allow for airflow while expiration was unimpeded. The breathing pattern was set to a prolonged duty cycle (0.7) at a breathing frequency of 15 breaths·min^−1^ while targeting 60% of their maximal P_DI_ in order to promote diaphragm fatigue. Maximal P_DI_ was determined from a maximal inspiratory maneuver performed from functional residual capacity against a semi‐occluded mouthpiece. Participants repeated this maneuver until a minimum of three P_DI_ values within 10% of each other were collected. The highest value of these three maneuvers was used to determine the target P_DI_ of the IPTL task. The goal was to create a tension‐time index of the diaphragm (TTI_DI_), calculated as the product of the inspiratory duty cycle and the ratio of the mean P_DI_ generated per breath to the maximal P_DI_ generated, exceeding 0.2 to impede blood flow (Bellemare et al., 1983; Buchler et al., 1985) and promote fatigue (Bellemare & Grassino, 1982). Participants were given auditory feedback in the form of a metronome with distinct inspiratory and expiratory tones to assist matching the required duty cycle and visual feedback in real time to target P_DI_. End‐tidal partial pressure of CO_2_ (P_ET_CO_2_) was monitored throughout the task (VacuMed model 17,630; CA, USA) and in the event of hypocapnia, defined as a decrease in P_ET_CO_2_ of >2 mmHg, a mixture of 5% CO_2_ (balance N_2_) was introduced into the inspiratory circuit via a mixing chamber to return P_ET_CO_2_ to resting levels. Heart rate was continuously recorded using a commercially available heart rate monitor (Polar H10, Polar Electro, Kempele, Finland). Dyspnea intensity was evaluated at rest, during every minute of IPTL, and at task failure. Dyspnea was evaluated using the 0–10 modified category‐ratio Borg scale (Borg, 1982), anchored such that “0” represented “no breathing discomfort at all” and “10” represented “the most intense breathing discomfort you have ever experienced or could imagine experiencing”. Participants were coached to adopt a diaphragmatic breathing pattern before beginning the IPTL task by breathing off the mouthpiece with one hand on their abdomen and another on their ribs with the goal of feeling the hand on the abdomen move while the hand on the ribs remained stationary (Ramsook et al., 2016). This was done to emphasize diaphragm use before beginning the IPTL task. Participants were familiarized to the IPTL task by performing brief (no more than 1 min) bouts of IPTL on visit 1 targeting P_MO_ as opposed to P_DI_ as no balloon catheters were used on these familiarization visits. No coaching or encouragement on the respiratory muscle activation pattern was given during the task as it would interfere with our ability to explore the role of respiratory muscle recruitment on diaphragm fatigability. Moreover, given established sex difference in respiratory muscle activation (Mitchell et al., 2018; Molgat‐Seon et al., 2018), we did not want to impose a breathing pattern that would disadvantage one sex over the other when performing the task. Task failure was defined as either (*i*) an inability to reach or maintain the target P_DI_ for the duration of the inspiration for three consecutive breaths or (*ii*) the participant felt they were no longer able to continue and voluntarily terminated the task.

### 2.6 Diaphragm neuromuscular function

Diaphragm muscle function was examined from two perspectives. First, we examined contractile function through the transdiaphragmatic twitch pressure (P_DI,TW_). Second, we examined the ability for an individual to generate maximal P_DI_ through D‐VA. A decrease in either metric was considered evidence of diaphragm fatigue. Cervical magnetic stimulation (CMS) was delivered by a magnetic stimulator (MagStim 200^2^, The MagStim Company Ltd., Whitland, Wales), along the third through seventh cervical vertebra, based on the location that resulted in the strongest individual P_DI,TW_ and CMAP to stimulate the phrenic nerves as previously described (Similowski et al., 1989). All stimuli delivered at rest were initiated at functional residual capacity, indicated by the participant giving a hand signal “at the end of a normal breath out” and later confirmed by checking the P_ES_. To determine whether CMS elicited a maximal stimulation of the phrenic nerves, a recruitment curve was developed for each participant by eliciting three unpotentiated twitches, initiated from functional residual capacity, at increasing stimulator output intensity (60%, 70%, 80%, 90%, 95%, and 100%). Stimulation was considered maximal for an individual participant if the difference in P_DI,TW_ between maximal and submaximal output intensities was less than the coefficient of variation for the measurement for each individual. Interpolated twitches were performed in blocks before IPTL and immediately after completing IPTL. Blocks consisted of 5–9 interpolated twitch maneuvers, and the first two twitches of each block were excluded from the analysis to ensure potentiation of each subsequent stimulation. The average of the first three acceptable twitches were used for analysis. Reasons for excluding twitch maneuvers included (*i*) the stimulation was not performed close to functional residual capacity, (*ii*) the twitch occurred as the participant was swallowing, (*iii*) cardiac artifact was superimposed on the CMAP, or (*iv*) the diaphragm was not relaxed, determined from EMG_DI_ activity (Laghi et al., 1996). Each interpolated twitch maneuver began with a maximal inspiratory effort from functional residual capacity against a semi‐occluded mouthpiece apparatus, during which a single twitch was delivered via CMS (superimposed twitch). Following this effort, a control twitch was delivered when the participant returned to functional residual capacity. This technique has previously been shown to be a reliable means of measuring D‐VA (Ramsook et al., 2021). Voluntary activation was calculated as follows: (Merton, 1954).

$$\begin{array}{c} \text{Voluntary Activation}\;\left(\%\right)=\\ \left[1–\left(\frac{\text{superimposed twitch}}{\text{control twitch}}\right)\right]\times 100\% \end{array}$$

Diaphragm contractile function was assessed by comparing the P_DI,TW_ amplitude of the control twitch before and after IPTL. Participants were given continuous encouragement throughout each interpolated twitch maneuver to ensure maximal effort.

### 2.7 Statistical analyses

Participant characteristics and baseline D‐VA were tested for normality using the Shapiro–Wilk test and compared between sexes using an independent samples Student's *t*‐test if data were normally distributed. Both time to task failure (TTF) and cumulative diaphragm pressure generation violated normality and were tested with the Mann–Whitney *U*‐test. These data are presented as median (interquartile range). A one‐way repeated measures ANOVA determined if CMS maximally stimulated the phrenic nerves in each group. P_DI,TW_ during the recruitment curve protocol at submaximal stimulator output intensities was compared against the P_DI,TW_ at 100% stimulator output intensity with Dunnett's *post hoc* test. Paired *t*‐tests were used to compare the difference in absolute P_DI,TW_ and D‐VA for each sex before and after IPTL. To assess differences in fatigability between sexes, an independent samples *t*‐test compared the postexercise change in P_DI,TW_ and D‐VA, expressed as a percent of the respective baseline values. A two‐way (time and sex) repeated measures ANOVA assessed differences in mechanical (contraction and half‐relaxation time) and electrical (CMAP amplitude, onset, and duration) twitch properties between males and females, and before and after exercise. To minimize multiple comparisons and ensure an equal number of observations at each time point, variables during IPTL, including EMG, heart rate, and dyspnea, were compared at baseline (*i.e.,* breathing on the apparatus with no resistance and freely chosen respiratory duty cycle), the first minute of IPTL and the last minute of IPTL using a repeated measures ANOVA. Simple linear regression tested the relationship between fatigability measures (D‐VA and P_DI,TW_ after IPTL) and select measures including TTF, cumulative diaphragm pressure generation, and the average EMG from the final stage of IPTL using the “lm” function in R (R Core Team, 2021). Statistical analyses were performed in R (v.4.1.0). A *p* value <0.05 was considered significant, and data are presented as mean ± SD unless otherwise stated. Individual data are presented in box‐and‐whisker plots along with median (thick line), first and third quartiles (top and bottom borders of boxes), and the respective quartiles plus (first) or minus (third) 1.5 times the interquartile range (whiskers).

## 3 RESULTS

### 3.1 Participants

A total of 12 healthy males and 15 females were recruited to participate in this study. During the data analysis phase, 2 males and 4 females failed to display a plateau in their individual CMS recruitment curve and were excluded from the analysis. As such, 10 males and 11 females were included in the final analysis. All participants had pulmonary function within normal limits (Table 1).

**TABLE 1:** Participant characteristics

	Female	Male
*N*	11	10
Age, years	23 ± 5	23 ± 3
Height, cm	164 ± 7	178 ± 6^a^
Mass, kg	62 ± 9	74 ± 9^a^
BMI, kg· m^−2^	23 ± 2	23 ± 3
Pulmonary function		
FVC, L (%predicted)	4.19 ± 0.97 (100 ± 16)	5.72 ± 0.74^a^ (101 ± 9)
FEV_1_, L (%predicted)	3.39 ± 0.65 (95 ± 13)	4.49 ± 0.62^a^ (95 ± 9)
FEV_1_/FVC (%predicted)	0.82 ± 0.08 (95 ± 8)	0.78 ± 0.05 (93 ± 6)
TLC, L (%predicted)	5.36 ± 1.14 (96 ± 13)	7.50 ± 1.04^a^ (101 ± 10)
MIP, cmH_2_O (%predicted)	98 ± 31 (106 ± 33)	126 ± 27^a^ (97 ± 21)

Abbreviations: BMI, body‐mass index; FEV_1_, forced expired volume in 1 s; FVC, forced vital capacity; MIP, maximal inspiratory pressure; TLC, total lung capacity.

^a^ *p* < 0.05. Values are mean ± SD.

### 3.2 Inspiratory pressure threshold loading

**FIGURE 1:**
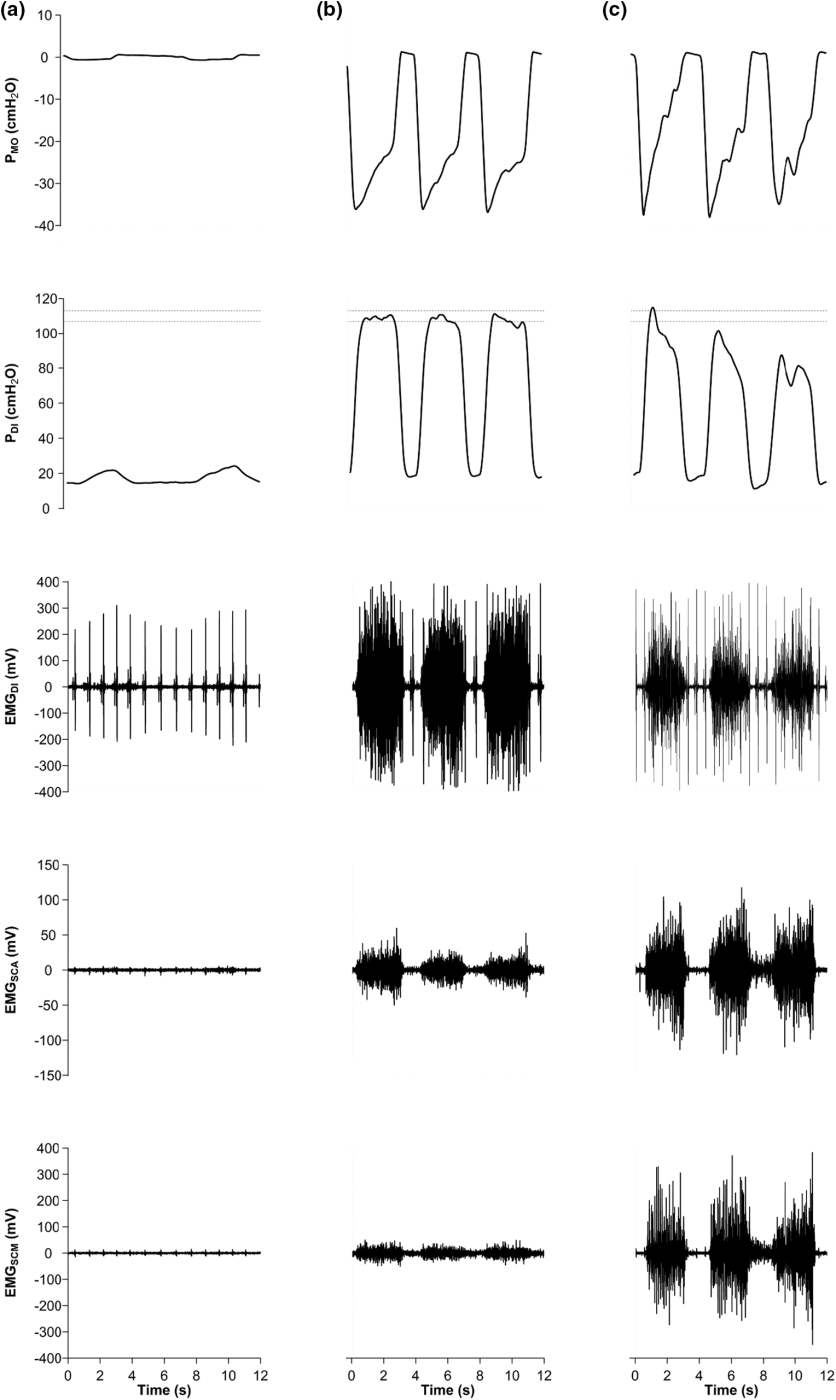
Example of IPTL protocol. Experimental data from a male participant performing IPTL while breathing on the IPTL device with no resistance (*i.e.*, baseline) (a), during the first minute of IPTL (b), and at task failure (c). Data from top to bottom are raw P_MO_, raw P_DI_ along with a dotted lines representing the target P_DI_ range, filtered EMG_DI_, EMG_SCA_, and EMG_SCM_. EMG_DI_, diaphragm electromyography; EMG_SCA_, scalene electromyography; EMG_SCM_, sternocleidomastoid electromyography; P_DI_, transdiaphragmatic pressure; P_MO_, mouth pressure

**FIGURE 2:**
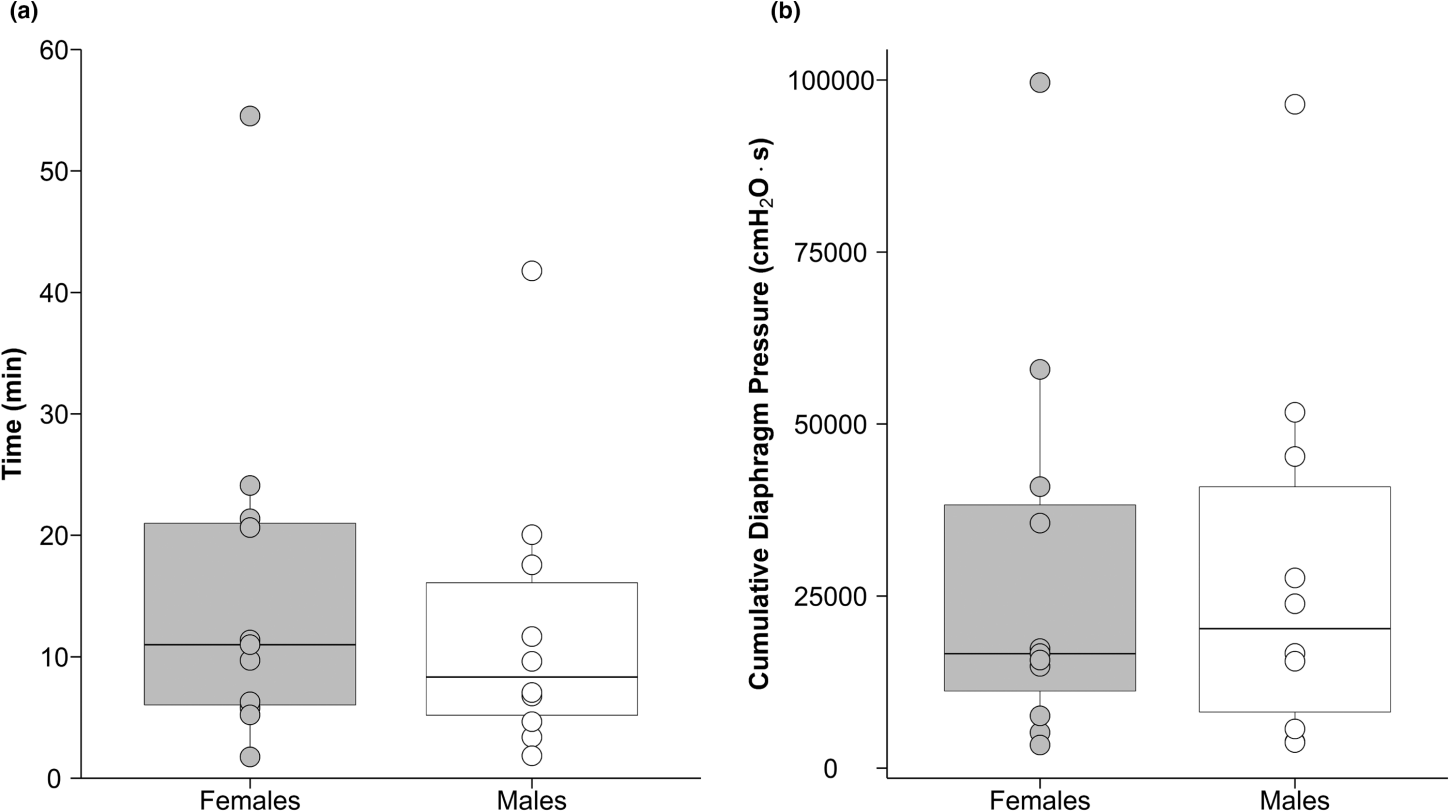
Box‐and‐whisker plot and individual data showing time to task failure (a) and cumulative diaphragm pressure output at task failure (b). Circles represent individual data; thick line inside each box represents the median; top and bottom borders of the box represent the third and first quartiles, respectively; and the whiskers of each box represent the first quartile plus 1.5 times the interquartile range and the third quartile minus 1.5 times the interquartile range for the top and bottom whisker, respectively. No significant differences between sexes were observed

An example of raw pressure and EMG data collected during IPTL in an individual participant is shown in Figure 1. Baseline maximal transdiaphragmatic pressure (P_DI,MAX_) was not different between sexes (F:127 ± 32, M: 139 ± 30 cmH_2_O, *p* = 0.410) despite males having greater absolute inspiratory muscle strength as assessed through maximal inspiratory pressure. Female participants, on average, targeted 76 ± 19 cmH_2_O and males targeted 83 ± 18 cmH_2_O (*p* = 0.418). At the start of the task, some participants (F: 2; M: 2) had difficulty maintaining the desired breathing frequency; however, they were able to adopt the correct breathing frequency as the task proceeded. During the final minute of IPTL, both pressure generation and breathing pattern began to deviate from the desired protocol. Nevertheless, a TTI_DI_ above 0.2 was present from the beginning of the task (F: 0.47 ± 0.08; M: 0.46 ± 0.05) and at the end of the task (F: 0.37 ± 0.06; M: 0.40 ± 0.07) and was maintained throughout the task for each participant. TTF was 11.0 (15.0) min in females and 8.3 (10.9) min in males (median (interquartile range); *p* = 0.557; Figure 2a). Four participants (3 female and 1 male) voluntarily terminated the task. P_ET_CO_2_ was maintained within 3 mmHg from initial values throughout IPTL. Additional cardiorespiratory variables measured at baseline, during the first minute of IPTL and at peak are presented in Table 2. Dyspnea increased throughout the IPTL task (main effect of time, *p* < 0.001) but was not different between sexes (*p* = 0.426). At the end of the IPTL task, the cumulative diaphragm pressure generation was not different between females (16,625 (27,062) cmH_2_O·s) and males (20,271 (32,719) cmH_2_O·s, median (interquartile range), *p* = 0.97; Figure 2b).

**TABLE 2:** Cardiorespiratory response during inspiratory pressure threshold loading

		Baseline	First minute	Peak
Time to task failure, min	Female	‐‐	‐‐	11.0 (15.0)
	Male	‐‐	‐‐	8.3 (10.9)
Heart rate, beat·min^−1^ ^a^	Female	68 ± 14	80 ± 15	93 ± 25
	Male	65 ± 12	89 ± 10	90 ± 7
SpO_2_, %^a^	Female	98 ± 1	97 ± 1	96 ± 2
	Male	97 ± 2	97 ± 1	95 ± 3
Dyspnea, 0–10 scale^a^	Female	0.1 ± 0.3	1.4 ± 1.6	7.3 ± 2.4
	Male	0 ± 0	1.9 ± 2.5	8.2 ± 2.3
f_b_, breath·min^−1^ ^a^	Female	15.0 ± 5.0	16.5 ± 1.5	20.3 ± 4
	Male	17.3 ± 4.8	16.0 ± 1.4	17.3 ± 2
T_I_, s^a^	Female	2.14 ± 0.71	2.91 ± 0.26	2.30 ± 0.50
	Male	2.18 ± 0.62	2.88 ± 0.26	2.77 ± 0.33
T_I_/T_TOT_ ^a^	Female	0.51 ± 0.17	0.80 ± 0.06	0.76 ± 0.08
	Male	0.59 ± 0.14	0.76 ± 0.04	0.79 ± 0.05

*Note*: Values are mean ± SD except for TTF which are presented as median (IQR).

Abbreviations: S_P_O_2_, peripheral oxyhemoglobin saturation; f_b_, breathing frequency; T_I_, inspiratory time; IQR, interquartile range; T_TOT_, total breath time.

^a^ Main effect of time (*p* < 0.05).

### 3.3 Inspiratory muscle electromyography during IPTL

**FIGURE 3:**
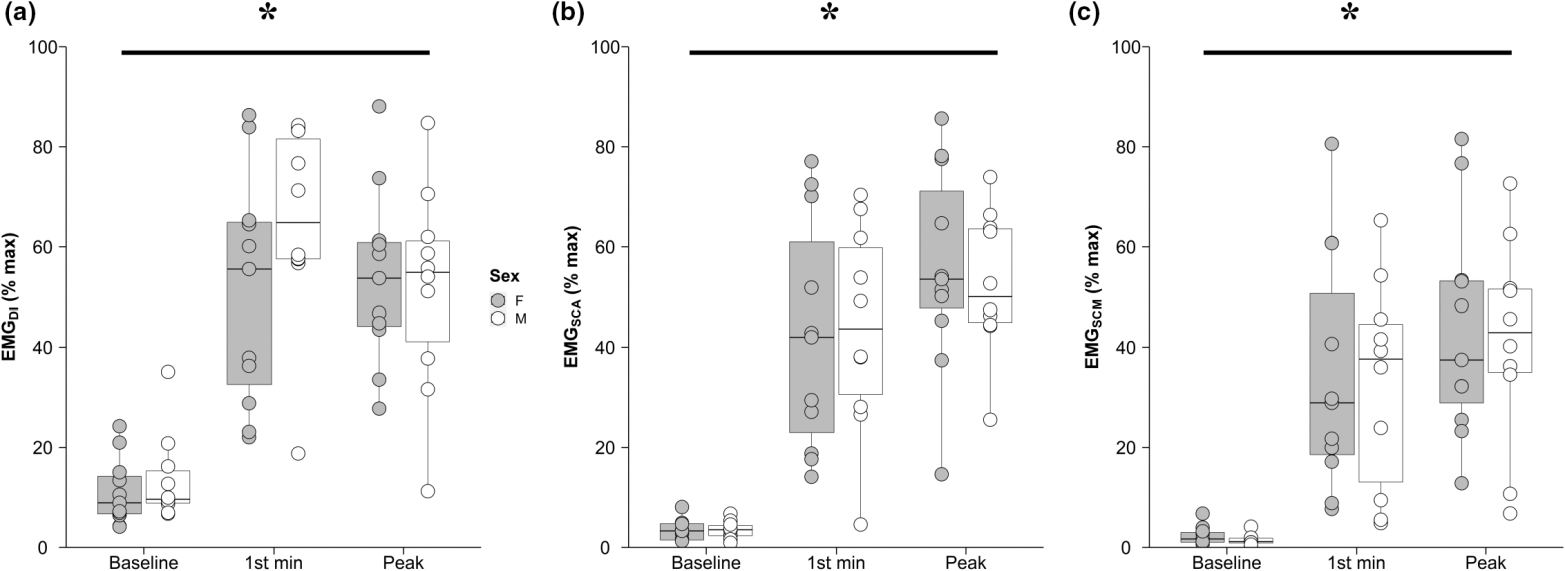
Inspiratory muscle electromyography. Box‐and‐whisker plot and individual data of EMG_DI_ (a), EMG_SCA_ (b) and EMG_SCM_ (c) at baseline, after the first minute of IPTL, and at task failure. Circles represent individual data; thick line inside each box represents the median; top and bottom borders of the box represent the third and first quartiles, respectively; and the whiskers of each box represent the first quartile plus 1.5 times the interquartile range and the third quartile minus 1.5 times the interquartile range for the top and bottom whisker, respectively. *, main effect of time (*p* < 0.05); EMG_DI_, crural diaphragm electromyography; EMG_SCA_, scalene muscle electromyography; EMG_SCM_, sternocleidomastoid muscle electromyography; F, females; IPTL, inspiratory pressure threshold loading; M, males

Box‐and‐whisker plots along with individual values for inspiratory muscle EMG are presented in Figure 3 and mean values in Table 3. A main effect of sex was not observed across all inspiratory muscles (all *p* > 0.05); however, a main effect of time was observed in EMG_DI_ (*p* < 0.001), EMG_SCA_ (*p* < 0.01), and EMG_SCM_ (*p* < 0.01). Extra‐diaphragmatic (EMG_SCM_ and EMG_SCA_) inspiratory muscle activity increased substantially from rest to the first minute of IPTL and continued to increase until task failure. EMG_DI_ increased at the start of the IPTL task and did not change at task failure (*p* = 0.325).

**TABLE 3:** Inspiratory muscle electromyography during inspiratory pressure threshold loading

		Baseline	First minute	Peak
EMG_DI_, %max^a^	Female	11.1 ± 6.7	51.3 ± 23.1	53.8 ± 17.4
	Male	13.6 ± 8.7	64.8 ± 20.0	51.7 ± 20.7
EMG_SCA_, %max^a^	Female	3.4 ± 2.1	42.1 ± 23.1	55.7 ± 20.4
	Male	1.4 ± 1.1	43.8 ± 20.7	52.8 ± 14.2
EMG_SCM_, %max^a^	Female	2.3 ± 1.8	34.2 ± 23.8	43.3 ± 21.7
	Male	1.4 ± 1.1	32.6 ± 21.0	41.2 ± 20.7

Abbreviations: EMG_DI_, crural diaphragm electromyography; EMG_SCA_, scalene electromyography; EMG_SCM_, sternocleidomastoid EMG.

^a^ Main effect of time (*p* < 0.05). Values are mean ± SD.

### 3.4 Diaphragm neuromuscular function

**FIGURE 4:**
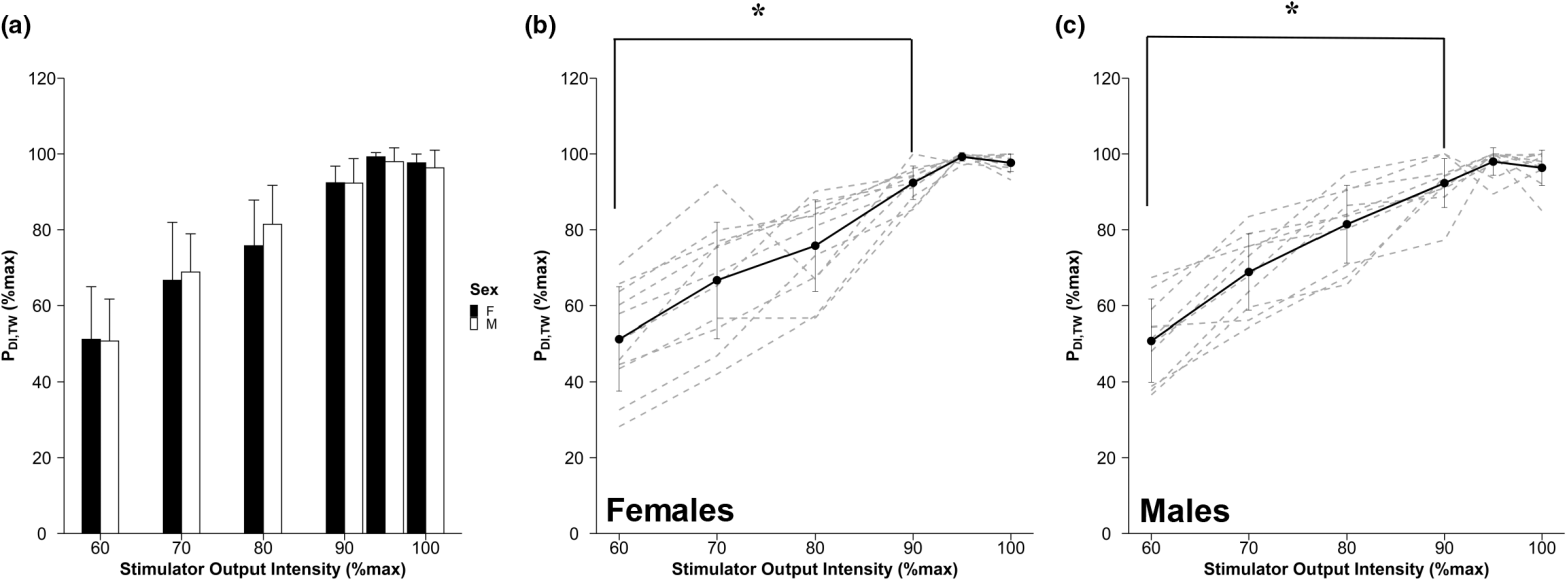
Transdiaphragmatic twitch response to increasing stimulator output intensity. Bar graph depicting P_DI,TW_ (mean ± SD) as a percent of max P_DI,TW_ in response to increasing intensities of the magnetic stimulator (a). Mean (solid line) and individual responses (dashed lines) to increasing stimulator output intensity in females (b) and males (c). P_DI,TW_ response did not significantly increase from 95% to 100%. P_DI,TW_, transdiaphragmatic twitch amplitude, *, significantly different from 100% (*p* < 0.05)

**FIGURE 5:**
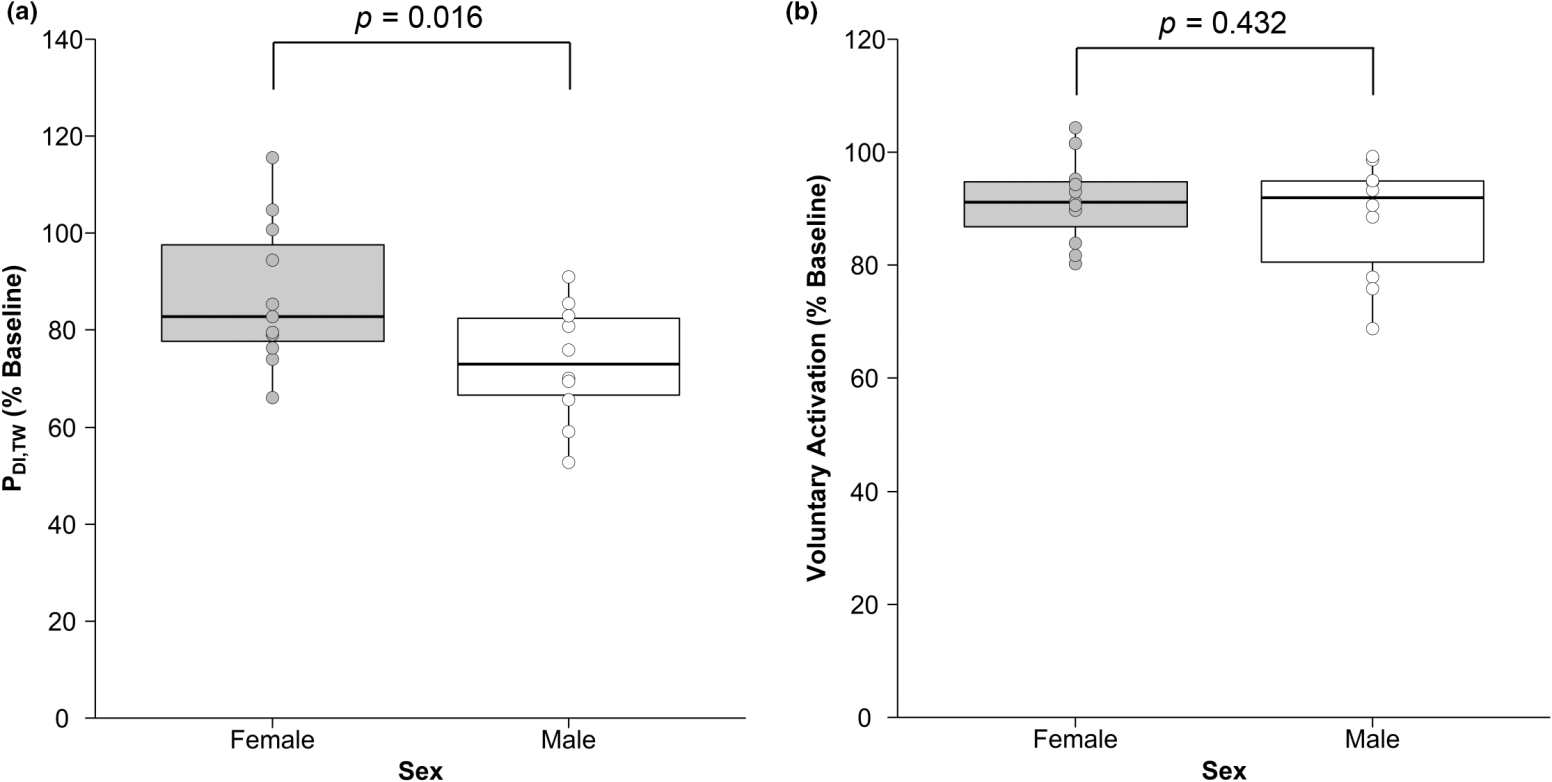
Diaphragm fatigability measures. Change in P_DI,TW_ (a) and change in D‐VA (b) after IPTL expressed as % of baseline. Circles represent individual data; thick line inside each box represents the median; top and bottom borders of the box represent the third and first quartiles, respectively; and the whiskers of each box represent the first quartile plus 1.5 times the interquartile range and the third quartile minus 1.5 times the interquartile range for the top and bottom whisker, respectively. P_DI,TW_, transdiaphragmatic twitch amplitude

In response to increasing stimulator output intensity, there was no significant increase in P_DI,TW_ between 95 and 100% (Figure 4). None of the participants presented with a P_DI,TW_ of ≤15 cmH_2_O, which would be indicative of diaphragm weakness (Laveneziana et al., 2019). Twitch maneuvers were performed 1.2 ± 0.4 min (range 0.6–2.0 min) after terminating the IPTL task. Individual P_DI,TW_ responses are presented in Figure 5a. A statistically significant loss in P_DI,TW_ was observed in females (36 ± 10 vs. 31 ± 8 cmH_2_O; pre vs. post; *p* = 0.008) and males (44 ± 12 vs. 33 ± 12 cmH_2_O; pre vs. post; *p* < 0.001). Similarly, a statistically significant decline in D‐VA was also observed in females (91 ± 7 vs. 83 ± 10%; pre vs. post; *p* = 0.002) and males (90 ± 4 vs. 80 ± 11%; pre vs. post; *p* < 0.001). At baseline, D‐VA was not different between sexes (F: 91 ± 7, M: 90 ± 4%, *p* = 0.683). After the IPTL task, P_DI,TW_ was significantly lower in males compared to females (73 ± 12 vs. 87 ± 15% baseline, respectively; *p* = 0.016). Both the contraction time (main effect of time, *p* = 0.002) and half‐relaxation time (main effect of time, *p* = 0.012) changed after IPTL but did not differ between sexes (both main effect of sex, *p* > 0.05). Additional mechanical outcomes associated with the P_DI,TW_ can be found in Table 4. When comparing the change in D‐VA following IPTL relative to baseline (Figure 5b), we observed no differences between females and males (91 ± 8 vs. 88 ± 10% baseline, respectively; *p* = 0.432).

**TABLE 4:** Diaphragm fatigability measures

	Female		Male	
	Baseline	Post‐IPTL	Baseline	Post‐IPTL
Contraction time, ms^a^	126.7 ± 8.2	122.9 ± 12.0	125.2 ± 9.3	118.8 ± 12.7
Half‐relaxation time, ms^a^	72.1 ± 9.2	67.3 ± 15.0	69.0 ± 10.3	64.3 ± 12.9
CMAP amplitude, mV	1.75 ± 0.65	1.58 ± 0.74	1.66 ± 0.72	1.61 ± 0.66
CMAP onset, ms	7.3 ± 1.3	6.7 ± 1.9	7.9 ± 0.9	7.6 ± 1.6
CMAP duration, ms	20.9 ± 3.0	19.9 ± 5.2	20.5 ± 1.9	20.0 ± 4.1

Abbreviations: IPTL, inspiratory pressure threshold loading; CMAP, compound muscle action potential.

^a^ Main effect of time (*p* < 0.05). Values are mean ± SD.

**FIGURE 6:**
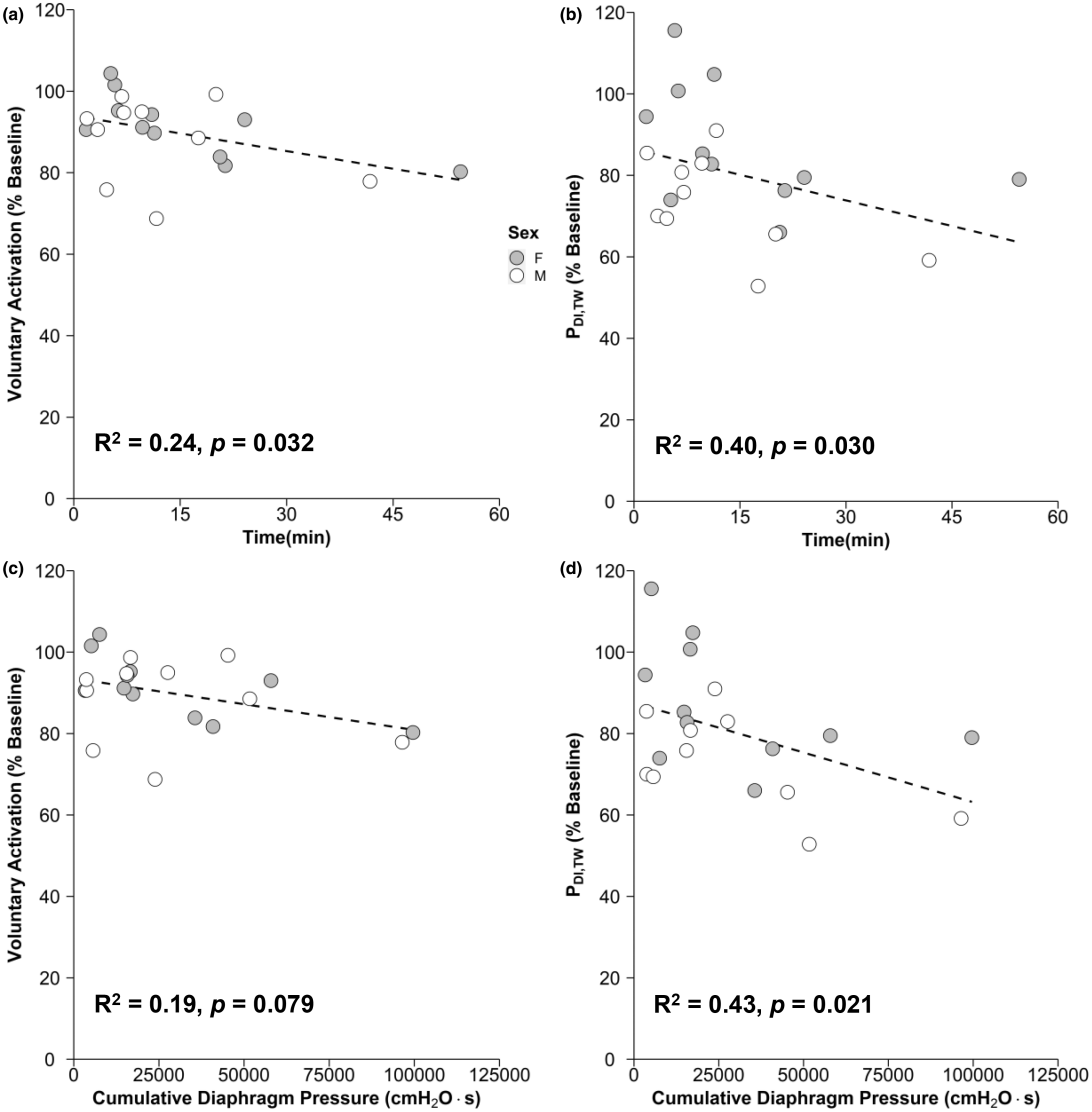
Relationship between diaphragm fatigability outcomes and IPTL. Scatter plot showing the relationship between D‐VA after IPTL and TTF (a), P_DI,TW_ and TTF (b), D‐VA after IPTL and cumulative diaphragm pressure output (c), and P_DI,TW_ after IPTL and cumulative diaphragm pressure output (d). Dashed line is a linear regression representing the relationship between the associated variables. D‐VA, diaphragm voluntary activation; IPTL, inspiratory pressure threshold loading; P_DI,TW_, transdiaphragmatic twitch amplitude

D‐VA (% baseline) after IPTL was significantly correlated with TTF (R^2^ = 0.24; *p* = 0.041; Figure 6a); however, D‐VA did not significantly correlate with cumulative diaphragm pressure generation (*R* ^2^ = 0.19; *p* = 0.079). In contrast, change in P_DI,TW_ was significantly correlated with both cumulative diaphragm pressure generation (*R* ^2^ = 0.43; *p* = 0.021; Figure 6b) and TTF (R^2^ = 0.40; *p* = 0.030). The relationship between EMG_SCM_ at task failure and D‐VA after IPTL was weak (*R* ^2^ = 0.19) and not significant (*p* = 0.080). A similar pattern was observed between EMG_SCA_ at task failure and D‐VA (*R* ^2^ = 0.19; *p* = 0.077). There was no notable relationship between EMG_DI_ at task failure and D‐VA (*R* ^2^ = 0.06; *p* = 0.409). There were no significant relationships between the change in P_DI,TW_ and EMG_DI_ (*R* ^2^ = 0.04; *p* = 0.738), EMG_SCA_ (*R* ^2^ = 0.02; *p* = 0.835), or EMG_SCM_ (*R* ^2^ = 0.00; *p* = 0.99) at task failure.

## 4 DISCUSSION

### 4.1 Main findings

The purpose of this study was to determine whether young, healthy adults would experience a decline in D‐VA after isolated, fatiguing inspiratory work. We also sought to explore whether the decline in D‐VA would differ between sexes. This study showed that both contractile and neural mechanisms contribute to diaphragm fatigability in males and females, measured by the change in P_DI,TW_ and D‐VA, respectively, during isolated inspiratory muscle loading. Contrary to our hypothesis, we did not observe a sex difference in the decline in D‐VA. The change in D‐VA was significantly correlated with TTF, whereas the change in P_DI,TW_ was correlated with cumulative diaphragm pressure generation and TTF. Collectively, these findings suggest that changes in D‐VA are more influenced by the time spent performing a task as opposed to the total respiratory pressure generation.

### 4.2 Diaphragm neuromuscular function

There was a loss in contractile function of the diaphragm following IPTL, as evidenced by a decrease in P_DI,TW_. We found the decrease in P_DI,TW_ to be moderately and significantly correlated to the cumulative diaphragm pressure generation. This is similar to the strong relationship observed between the decrease in P_DI,TW_ and cumulative diaphragm pressure generation during whole‐body exercise reported previously (Archiza et al., 2018). It is worth noting that our observation was independent of sex whereas the previous report only included male participants. In the present study, the decrease in P_DI,TW_ was greater in males compared to females despite generating similar amounts of relative and absolute transdiaphragmatic pressure, providing further evidence to support greater diaphragm fatigue resistance, as it relates to contractile function, in females compared with males. This finding is contrary to a previous study conducted where male and female participants, matched for absolute diaphragm strength, performed 5 min of IPTL in normoxia at a fixed intensity, and showed a similar decrease in P_DI,TW_ between sexes (Archiza et al., 2021). The discrepancy between the present study and the work of Archiza et al. (2021) may be explained by the fact that Archiza et al. (2021) had participants perform IPTL for a fixed duration instead of performing IPTL to task failure. This resulted in less variation in total inspiratory work and therefore may have resulted in a more uniform P_DI,TW_ response compared to our open task with a more variable P_DI,TW_ response after IPTL. It is possible that motivation could have influenced the TTF in the present study. No verbal encouragement was offered to participants during the IPTL task to maximize task standardization and avoid any bias from the experimenter who was aware of the study hypotheses. Additionally, despite familiarizing participants to the IPTL task, some participants still had difficulty in maintaining the required breathing pattern. In particular, one female participant was unable to match the desired breathing frequency after several minutes of IPTL and therefore met the objective criteria for task failure. This participant had an increase in P_DI,TW_ (115% baseline) and D‐VA (105% baseline) after IPTL. It is possible this participant did not perform enough respiratory work to incur any loss of P_DI,TW_ or D‐VA and perhaps her difficulty in meeting the required breathing pattern resulted in premature termination of the IPTL task, further supported by her relatively low rating of dyspnea at TTF (3 on the 0–10 Borg scale). In the present study, three female participants and one male participant voluntarily ended the IPTL task rather than approach task failure using our objective criteria. Unfortunately, the small sample size precludes us from making any statistical comparisons between those who did and did not achieve our objective criteria for task failure. Nevertheless, we observed that the participants who voluntarily ended the task tended to have lower than average dyspnea ratings at TTF (range: 3–6 on the 0–10 Borg scale). A larger sample is required to explore differences between these two groups. However, excluding the participants who voluntarily terminated the IPTL task did not result in a significantly different TTF or cumulative diaphragmatic pressure generation between sexes. Moreover, while similar relative intensities were used between studies, the absolute P_DI,MAX_, and subsequent target intensity for performing IPTL, was greater in the study by Archiza et al. (2021). While the absolute P_DI,MAX_ values were lower in our participants compared to previous studies (Archiza et al., 2021; Geary et al., 2019), the key component of the IPTL protocol was to create a task with a TTI_DI_ greater than 0.2 in order to promote a fatigable state (Bellemare & Grassino, 1982). In our study, we achieved this goal (TTI_DI_ of 0.47 ± 0.08 for females and 0.46 ± 0.05 for males). These two factors (*i.e.*, individual time to task failure and absolute inspiratory work performed) may explain the observed differences in the P_DI,TW_ response after IPTL between the two studies and explain the higher variability in TTF and fatigability outcomes among our participants. Nevertheless, these disparate results highlight the fact that task failure and overall performance fatigability are not governed by a single mechanism, but rather a host of factors, which work together to influence muscular performance.

D‐VA decreased in both males and females after IPTL. The decrease in D‐VA was significantly, albeit weakly, correlated with TTF when data were pooled together. A decrease in voluntary activation after isolated respiratory work has been reported previously after both inspiratory (Luu et al., 2020) and expiratory efforts (McKenzie et al., 1992) in male participants. To our knowledge, no previous studies have reported changes in D‐VA in females after IPTL. Interestingly, we did not observe a sex difference in the change in D‐VA after IPTL. This differs from our previous findings using whole‐body exercise where males experienced a greater loss of D‐VA after cycling exercise than females (Ramsook et al., 2022). This finding did not support our hypothesis. We hypothesized that the apparent blunting of the inspiratory muscle metaboreflex in females compared to males, as evidenced through an attenuated cardiovascular response to inspiratory muscle work (Geary et al., 2019; Smith et al., 2016; Welch et al., 2018a), could offer similar protection against a loss of D‐VA. Interestingly, the lack of sex difference in D‐VA occurred in the presence of a sex difference in the change in P_DI,TW_. This observation likely suggests that these different aspects of fatigability are mediated through different mechanisms. As alluded to earlier, feedback from group III/IV afferents can influence voluntary activation of the quadriceps (Sidhu et al., 2017), and may similarly influence D‐VA. However, contractile function may be maintained through mechanisms such as perfusion and the ability to clear fatigue‐inducing metabolites. While yet to be explored in humans, evidence from anesthetized female rabbits show greater diaphragm blood flow in response to an increase in ventilation compared to male rabbits (Lublin et al., 1995). It is possible that greater diaphragm blood flow may work to mitigate the loss of contractile function in females but have no effect on the loss of D‐VA. However, this remains speculative, and more studies are clearly needed to determine the precise physiological basis for sex differences in the various components of diaphragm fatigability. A lack of sex difference in D‐VA after IPTL could also be explained by task specificity. For example, while whole‐body exercise can result in a loss of contractile function, mimicking the hyperpnea of exercise does not result in a similar decrease in P_DI,TW_ (Babcock et al., 1995). The results from Babcock et al. (1995) show that respiratory work alone was insufficient to elicit a loss in diaphragm contractile function; however, the interaction between respiratory and locomotor muscle work experienced during whole‐body exercise was enough to negatively affect diaphragm function. This phenomenon has been observed by blocking afferent feedback via intrathecal fentanyl injection, which preserved quadriceps voluntary activation compared to exercise where afferent feedback was undisturbed (Sidhu et al., 2017). Perhaps without the added stimulus of additional afferent activity from the locomotor muscles, as is present during exercise, the total sympathetic activity was not sufficient to promote a sex difference in D‐VA after IPTL.

### 4.3 Inspiratory muscle electromyography

When performing bouts of inspirations that require the participant to generate substantial negative pressures, such as during inspiratory muscle training, participants will often preferentially recruit extra‐diaphragmatic inspiratory muscles (Ramsook et al., 2016). In an effort to encourage participants to target the diaphragm during IPTL, they were instructed to engage in a diaphragmatic breathing pattern to reach their target P_DI_. They were given no further instruction during the IPTL to explore whether distinct respiratory muscle recruitment patterns emerged between males and females. During resting breathing, the intercostal muscles of the rib cage contribute more to breathing in females compared to males, possibly owing to the shape of the rib cage (Bellemare et al., 2003). Moreover, during whole‐body exercise, females tend to engage extra‐diaphragmatic inspiratory muscles, such as the scalenes and sternocleidomastoids, to a greater degree than their male counterparts (Mitchell et al., 2018; Molgat‐Seon et al., 2018). We did not make a similar observation in the present study during IPTL as EMG_SCM_ and EMG_SCA_ were similar between the sexes.

The discrepancy in the observed sex difference in changes in D‐VA between the present study using IPTL and our previous work using whole‐body exercise (Ramsook et al., 2022) is likely attributable to differences in the task. IPTL requires that participants generate and sustain relatively large negative pressures at a fixed breathing frequency and duty cycle that are not replicated during exercise or other activities of daily living. However, IPTL has the advantage of isolating the respiratory muscles compared to whole‐body exercise, where locomotor and respiratory muscles must compete for a finite cardiac output. It is possible that as the diaphragm loses its ability to contract through neural mechanisms, the extra‐diaphragmatic inspiratory muscles are activated to a greater degree to achieve the desired negative pressure. Another potential explanation for the relationship between inspiratory muscle activation and diaphragm fatigue is that individuals who naturally generate the negative pressures associated with ITPL by using their extra‐diaphragmatic inspiratory muscles, do not impose the same demands on the diaphragm that would result in fatigue. It is possible that the present study was underpowered to adequately explore the relationship between D‐VA and inspiratory muscle activity, and future studies are warranted to investigate the role of respiratory muscle activation and fatigability.

### 4.4 Methodological considerations

The open‐ended nature of our IPTL task resulted in a wide range of TTF and therefore cumulative P_DI_. This variability in inspiratory work performed may have led to increased variability in the P_DI,TW_ and D‐VA response to IPTL. While a closed task with a fixed time limit and uniform inspiratory load may have resulted in a more consistent measure of fatigability, we would have not been able to explore relationships between TTF and cumulative pressure generation with changes in the neuromuscular function of the diaphragm. Future studies with various IPTL protocols, including lower intensity for longer durations as well as fixed duration trials, can provide new insight into the mechanisms of diaphragm fatigue.

The phrenic nerves control the diaphragm (Lane, 2011) and can be stimulated using electrical stimulation, transcranial magnetic stimulation, bilateral anterolateral magnetic stimulation, and CMS. One major difference between electrical stimulation and CMS is the output intensity of the stimulator. With electrical stimulation, the output intensity can be progressively increased to ensure maximal stimulation of the phrenic nerves; however, this method is not without limitations (Similowski et al., 1989). With CMS, the maximum output intensity cannot exceed the 100% threshold imposed by the stimulator. The fixed output means there is a chance that a stimulation of 100% is not sufficient to maximally stimulate the phrenic nerves. Despite this limitation, previous studies in our laboratory (Boyle et al., 2020) and others (Verges et al., 2006; Welch et al., 2018a) have shown that the majority of participants show maximal stimulation as evidenced by a plateau in the P_DI,TW_ or CMAP amplitude when progressively increasing stimulator output. CMS has been used to measure D‐VA in previous studies (Similowski et al., 1996) and we have shown this to be a reliable technique both within‐ and between‐session (Ramsook et al., 2021). Potential submaximal stimulations are an important limitation to the CMS technique, and perhaps, the use of electrical stimulation may provide unique insight into diaphragm fatigability. Nevertheless, we attempted to minimize this limitation of CMS by only including participants who showed a plateau in P_DI,TW_ in response to CMS.

Another limitation is that tidal volume and therefore minute ventilation were not measured during the IPTL task. While tidal volume was not necessary to the IPTL task, it nevertheless may have provided some further information regarding the breathing pattern adopted during IPTL.

A validated measure of muscle sympathetic activity would have allowed us to explore the relationship between the inspiratory muscle metaboreflex and the change in D‐VA. The inspiratory muscle metaboreflex is thought to be mediated by the accumulation of fatigue‐inducing metabolites in response to respiratory muscle work (Sheel et al., 2018). It has also been proposed that the metaboreflex responses in peripheral muscle can result in an inhibition of central motor drive (Amann, 2011). We were unable to measure sympathetic activity during IPTL via muscle sympathetic nerve activity or a surrogate such as mean arterial pressure. Future studies are needed to determine whether there is a relationship between sympathetic activity and D‐VA.

We did not limit testing of female participants to a specific phase of the menstrual cycle and did not exclude females currently taking oral contraceptives or using an intrauterine device. While menstrual cycle phase may have an influence on baseline voluntary activation of the knee extensor muscles, there does not appear to be an effect on the change in voluntary activation of the knee extensors after a fatiguing task (Ansdell et al., 2019).

We are limited in our interpretation of the neural control of the diaphragm. Our measurements of EMG can be used as a surrogate for respiratory drive; however, precise neural control of the diaphragm and other inspiratory muscles can be assessed by investigating the behaviors of individual motor units (Butler et al., 2014), frequently accomplished through fine‐wire or intramuscular needle electrodes. While this was not an outcome of interest to this study, we acknowledge that our inspiratory muscle EMG data reflect the relative activation of these muscles and not respiratory drive *per se*.

The dearth of existing data surrounding changes in D‐VA and sex differences precluded us from making an accurate a priori sample size calculation to explore sex differences in D‐VA. We hope the results from this study can provide a basis for power analyses to guide future research exploring the effects of D‐VA between the sexes.

### 4.5 Future directions

The results of this study provide a foundation for future studies to better understand diaphragm fatigability in males and females. Notably, the precise site where diaphragm fatigue occurs remains unknown. Future work is required to identify these sites, and once identified, target these sites to mitigate fatigue and potentially improve exercise performance. These studies may benefit from determining spinal and supraspinal sites that can affect diaphragm neuromuscular function. Furthermore, the impact of diaphragm fatigue on the multidimensional nature of dyspnea may lead to targeted therapies to improve outcomes in patients with respiratory disease. Collectively, these proposed areas of research will likely provide novel insight into the functional consequences of diaphragm fatigue. When examining the mechanisms contributing to changes in diaphragm neuromuscular function, future studies are needed to determine the role of sympathetic activity in the loss of D‐VA after fatiguing tasks and to further explore whether different extra‐diaphragmatic inspiratory muscle recruitment patterns can influence diaphragm fatigue. Finally, future studies are needed to understand the mechanisms through which whole‐body exercise affects diaphragm fatigability differently than isolated inspiratory muscle work.

## 5 CONCLUSIONS

In summary, we have shown that D‐VA decreases after a single bout of fatiguing inspiratory work in healthy humans. This study includes a novel investigation into sex differences in D‐VA after isolated inspiratory work. Both males and females experienced a similar decrease in D‐VA, despite males showing a greater loss in P_DI,TW_ compared to females after performing IPTL at similar relative and absolute intensities. These findings support some previous findings that the female diaphragm is less fatigable than males; however, our results also suggest that the loss in contractile function and voluntary activation are governed by unique processes. Finally, our observed sex‐similarity in the decrease in D‐VA is contrary to earlier findings of a sex difference in D‐VA after high‐intensity whole‐body exercise. Together, these findings reiterate that fatigability is task specific with potentially different underlying mechanisms. We speculate that D‐VA may have a greater impact on low‐to‐moderate intensity activity performed over a long period of time compared to high intensity and short duration activity; however, more work is required to confirm this.

## AUTHOR CONTRIBUTIONS

AHR and JAG conceived the study. AHR, MRS, RAM, SSD, KMM, and ONF collected the data. AHR, JHP, and JAG analyzed the data. All authors contributed to interpretation of the data and preparing and review of the final manuscript.

## FUNDING INFORMATION

This work was funded by a Discovery Grant from the Natural Sciences and Engineering Research Council (NSERC) of Canada. AHR was supported by a postgraduate scholarship from the NSERC, a University of British Columbia (UBC) 4‐Year Fellowship (UBC4YF), and the British Columbia Lung Association Respiratory Rehabilitation Fellowship (BCLA‐RRF). RAM was supported by a postgraduate scholarship from the NSERC, a UBC4YF, and BLCA‐RRF. MRS was supported by a Michael Smith Foundation for Health Research (MSFHR) Postdoctoral Fellowship. KMM was supported by a Michael Smith Health Research BC Postdoctoral Fellowship and a Transition to Faculty Award from The University of British Columbia Faculty of Medicine Academic Enhancement Fund. JAG was supported by a Canadian Institutes of Health Research Clinical Rehabilitation New Investigator Award and a Scholar Award from the MSFHR.

## ETHICAL APPROVAL

Written informed consent was obtained from all participants prior to testing. All experimental procedures were approved by The University of British Columbia and Providence Health Care Research Institute Ethics Board (UBC‐PHC REB number: H19‐00725) and conformed to the *Declaration of Helsinki*, apart from registration in a database.

## Supporting information


Data S1
