# Supplementary material for: Voluntary activation of the diaphragm after inspiratory pressure threshold loading
Source: Physiol Rep. 2023 Jan 25;11(2):e15575. doi: 10.14814/phy2.15575 (PMC9875816; doi:10.14814/phy2.15575)
Supplement: Supplementary file 1 — Data S1 [file PHY2-11-e15575-s001.pptx]

## Slide 1
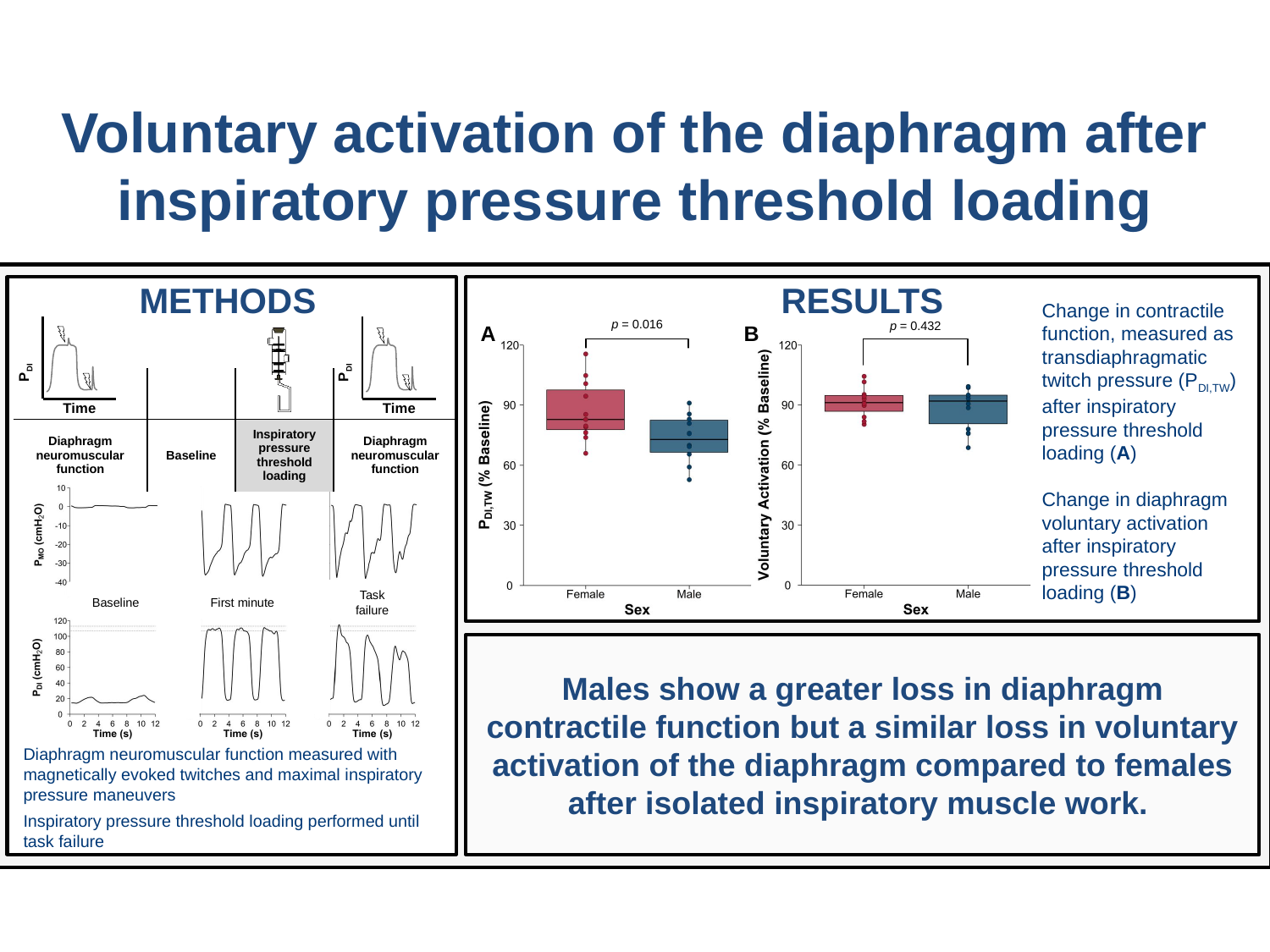

# Voluntary activation of the diaphragm after inspiratory pressure threshold loading
METHODS
RESULTS
Change in contractile function, measured as transdiaphragmatic twitch pressure (PDI,TW) after inspiratory pressure threshold loading (A)
Change in diaphragm voluntary activation after inspiratory pressure threshold loading (B)
p = 0.016
p = 0.432
A
B
PDI
Time
PDI
Time
| | | | |
| --- | --- | --- | --- |
| Diaphragm neuromuscular function | Baseline | Inspiratory pressure threshold loading | Diaphragm neuromuscular function |
First minute
Task failure
Baseline
Males show a greater loss in diaphragm contractile function but a similar loss in voluntary activation of the diaphragm compared to females after isolated inspiratory muscle work.
Diaphragm neuromuscular function measured with magnetically evoked twitches and maximal inspiratory pressure maneuvers
Inspiratory pressure threshold loading performed until task failure
